# Priming nanoparticle-guided diagnostics and therapeutics towards human organs-on-chips microphysiological system

**DOI:** 10.1186/s40580-016-0084-8

**Published:** 2016-10-01

**Authors:** Jin-Ha Choi, Jaewon Lee, Woojung Shin, Jeong-Woo Choi, Hyun Jung Kim

**Affiliations:** 1 Department of Biomedical Engineering, The University of Texas at Austin, Austin, TX 78712 USA; 2 Department of Chemical & Biomolecular Engineering, Sogang University, Seoul, 04107 Republic of Korea; 3 Interdisciplinary Program of Integrated Biotechnology, Sogang University, Seoul, 04107 Republic of Korea; 4 School of Medicine, Pusan National University, Yangsan, 50612 Republic of Korea

**Keywords:** Nanoparticle, Nano-biotechnology, Organs-on-chips, Microphysiological system, Diagnostics, Therapeutics

## Abstract

Nanotechnology and bioengineering have converged over the past decades, by which the application of multi-functional nanoparticles (NPs) has been emerged in clinical and biomedical fields. The NPs primed to detect disease-specific biomarkers or to deliver biopharmaceutical compounds have beena validated in conventional in vitro culture models including two dimensional (2D) cell cultures or 3D organoid models. However, a lack of experimental models that have strong human physiological relevance has hampered accurate validation of the safety and functionality of NPs. Alternatively, biomimetic human “Organs-on-Chips” microphysiological systems have recapitulated the mechanically dynamic 3D tissue interface of human organ microenvironment, in which the transport, cytotoxicity, biocompatibility, and therapeutic efficacy of NPs and their conjugates may be more accurately validated. Finally, integration of NP-guided diagnostic detection and targeted nanotherapeutics in conjunction with human organs-on-chips can provide a novel avenue to accelerate the NP-based drug development process as well as the rapid detection of cellular secretomes associated with pathophysiological processes.

## Introduction

Nanoparticles (NPs) have been extensively applied to biomedical fields. For example, gold NPs have specific optical properties and surface plasmon resonance (SPR) effect, which enable to rapidly and accurately detect biomarkers in combination with immunoselective molecules such as antibodies [1]. NPs perform as a therapeutic adjuvant with multiple functionality, by which programmed drug delivery as well as enhanced therapeutic efficacy can be expected [2]. Despite of these advantages, there are evident challenges in terms of the safety assessment of NPs in the human body [3, 4]. For instance, only a limited number of nanotherapeutics using albumin NPs [5] or iron oxide NPs [6] has been approved by the US Food and Drug Administration (FDA) because of the unclear pharmacokinetics (PK) of NPs in terms of the nanotoxicity and absorption, distribution, metabolism, and excretion (ADME) profiles [5]. To validate PK profiles of NPs, animal models (mostly rodent surrogates) have been widely exploited [7]. Indeed, the potential of animal models for testing NPs has been validated in a variety of biomedical researches such as diagnostics and therapeutics [2, 8–10], delivery of drugs or genes [11–13] and imaging for a target organ or transplanted cells [14, 15]. Furthermore, animal models have provided valuable in vivo rationales to ultimately target human, such as image-guided surgery using near infrared (NIR) emitting dye-loaded NPs [16], NP-based tracking of transplanted stem cells under magnetic resonance imaging (MRI) or fluorescence imaging [15], and cancer treatment using anti-cancer drug-conjugated NPs [17]. Although animal models are useful to track the translocation of NPs in vivo, it is notably challenging to understand cellular and molecular mechanism of NPs in a spatiotemporal manner. Moreover, the discrepancy in physiological responses between animal models and human may result in serious misunderstanding of the efficacy and nanotoxicity of NPs.

Alternatively, microfluidic models in part allow in vivo relevant culture of human cells under physiological fluid shear stresses mimicking either the blood circulation or the interstitial extracellular fluid that may influence on the transport of NPs [18, 19]. In recent years, breakthroughs of microfluidic approaches in combination with multi-cellular co-culture have emerged biomimetic human “Organs-on-Chips” microphysiological system that can recapitulate the in vivo relevant physiology and pathology in vitro [20]. The microengineered human organ models have leveraged the “Reverse Engineering” approach to demonstrate organ-level responses of highly organized tissue surrogate reconstituted in a structurally defined 3D microfluidic device under organ-specific mechanical actuations [21]. In this bioinspired organomimetic model, multiple types of tissue-specific human cells including differentiated epithelium, capillary or lymphatic endothelium, mesenchymal connective tissues, tissue-resident or circulating immune cells, as well as living human microbiome can be contemplated to cogently validate the efficacy and toxicity of NP-based nanotherapeutics.

In this review, we discuss recent progresses of NP-guided diagnostics and therapeutics that have been applied to the in vitro models. In particular, we focus on the potential applications of NPs in human organs-on-chips microsystems to assess the functional reactivity and the detection capacity of NPs that can be translated to applicability in clinical diagnostics and therapeutics.

## In vitro models for validating the functionality of NPs

### Application of the NPs in the biomedical field

NPs are materials with a size that range between 1 and 1000 nm. NPs have been applied in chemical, physical, biological, environmental, pharmaceutical, biomedical, and clinical fields based upon the physicochemical and photonic properties [7, 22]. NPs have strong reactive nature in chemical and biological reactions due to the high surface-to-volume ratio [23], which enables versatile applicability in in vivo imaging and clinical therapeutics. Inorganic NPs made of gold or magnetics have been used for the SPR effects [1] or magnetic resonance [24], respectively. Organic NPs such as biodegradable polymeric NPs [25] or liposomes [26] have been used for drug encapsulation. It is technically straightforward to conjugate biological or chemical drugs with diverse functional moieties displayed on the NPs [27]. For example, monoclonal antibodies (mAb) can be conjugated to the surface of NPs to detect specific biomarkers or receptors expressed on the cell surface. Various fluorescent materials such as fluorescein isothiocyanate (FITC) or green fluorescent protein (GFP) have been employed to encapsulate the NPs for imaging purpose [28].

**Fig. 1:**
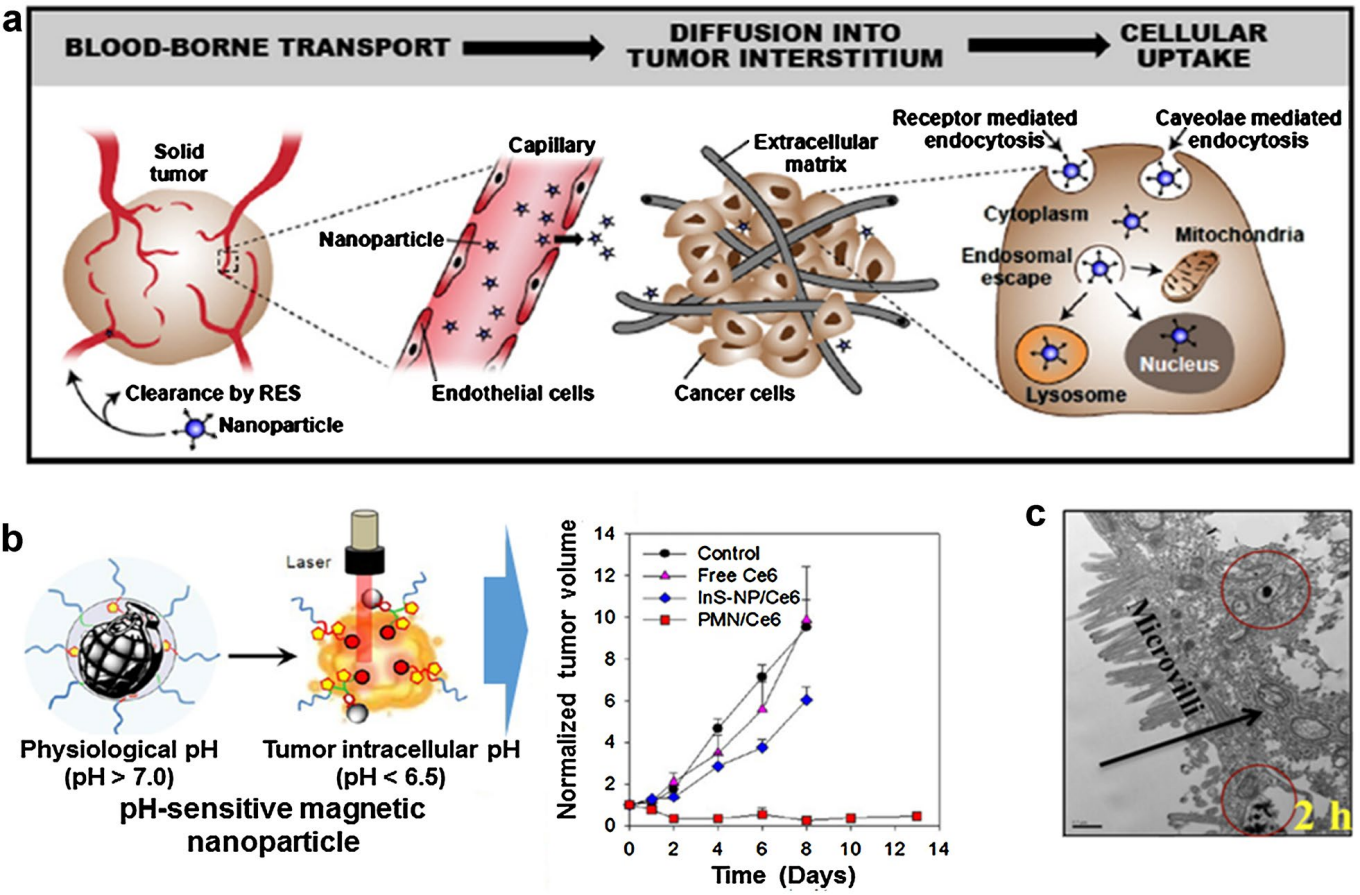
Therapeutic applications of NPs. **a** Mechanism of the transport of NPs to the tumor lesion through blood vessels. NPs escape reticuloendothelial system (RES) and reach to the targeted tumor cells. NPs present enhanced permeability and retention (EPR) during the delivery through the vascular system. The targeting ligand-modified NPs can specifically bind to the receptors on the surface of cancer cells. Reproduced with permission [29]. Copyright 2014, Elsevier. **b** Multifunctional pH-sensitive NPs consists of 3 nm iron oxide NPs (average diameter, ~70 nm) and pH-responsive ligand induce charge repulsion in pH below 6.5. NPs-guided photodynamic therapy is demonstrated to the human colon cancer (HCT116) cells by displaying magnetic resonance imaging after laser irradiation. A *graph* shows that the pH sensitive NP-therapeutics successfully inhibit the growth of tumor. Reproduced with permission [34]. Copyright 2014, American Chemical Society. **c** Internalization of the gold NPs (50 nm in diameter) into intestinal Caco-2 epithelial cells. After 2 h of treatment, gold NPs indicated by red circles penetrate through the microvilli on the apical brush border (*a white dotted oval circle*) of Caco-2 cells. An *arrow* indicates the direction of cellular uptake from apical to basolateral side across microvilli. *Scale bar* 500 nm. Reproduced with permission [39]. Copyright 2015, American Chemical Society

NPs have several advantages for the applications of in vivo diagnostics, imaging, and therapeutic treatment [2]. NPs can be leveraged for the targeted delivery to the tumor region for localized distribution and facilitated cellular uptake. NPs can specifically navigate to tumor cells in a passive or an active way (Fig. 1a) [29]. Intravenously injected NPs are passively delivered to the tumor region, where the surrounding neovascularized blood vessels have abundant proliferating endothelial cells, deficient pericytes, and aberrant basement membrane formation, which leads to the increased vascular permeability [30]. Moreover, lymph vessels around the tumors do not efficiently function to drain body fluid from the tumor region. Thus, the NPs loaded with drugs present an enhanced permeability and retention (EPR) effect of the drugs during the delivery through the vascular system [30]. NPs conjugated with aptamers or antibodies can specifically bind to the receptors on the surface of cancer cells such as vascular endothelial growth factor receptor (VEGFR), a key mediator of angiogenesis [31]. Since the healthy non-cancerous cells have significantly less expression of VEGFR, the aptamer-conjugated NPs penetrate through the cancer cells more efficiently via receptor-mediated endocytosis. NPs can be also used as a delivery carrier of drug compounds with enhanced stability [32], by which the controlled release of carried drugs triggered by pH, temperature, or enzymatic reactions is possible to the target site [33]. For example, pH-sensitive magnetic nano-complex composed of iron oxide NPs and pH-responsive ligand demonstrates effective diagnosis and therapy against colorectal cancer. By targeting the acidic tumor microenvironment, pH-responsive NPs can generate singlet oxygen in the tumor, which induces photodynamic therapeutic reactions to selectively kill cancer cells (Fig. 1b) [34]. NPs can also have photothermal effects in combination with therapeutic effects of embedded drugs [35, 36], which synergistically boosts up the efficacy of clinical interventions.

### Static 2D cell culture models

Existing 2D cell culture models are mostly static, simple, and straightforward to carry out the assessment of the cytotoxicity of NPs. After treating various cell lines with NPs for 2–72 h, trypan blue assay, fluorescence-based viability assay using a mixture of Calcein AM (live) and ethidium homodimer-1 (dead), lactate dehydrogenase (LDH) assay, or 3-(4,5-dimethylthiazol-2-yl)-2,5-diphenyl tetrazolium bromide (MTT) assay have been abundantly applied to test the cytotoxicity of NPs [37, 38]. For example, permeability and cytotoxicity of NPs are analyzed using a polarized human intestinal epithelial Caco-2 monolayer grown on the Transwell [39]. In this study, three different sizes of gold NPs (15, 50 and 100 nm in diameter) are applied, in which the transport of NPs through a Caco-2 monolayer is measured via inductively coupled plasma mass spectroscopy (ICP-MS) (Fig. 1c). NPs with 50 nm in diameter show the highest apparent permeability for various time periods (up to 24 h). This study reveals that the size of NPs may influence on the absorption, accumulation, and nanotoxicity of NPs to the intestinal epithelium. However, the 2D cell culture models are too simple to emulate the complex in vivo human microenvironment because available cell lines often poorly differentiate [40]. Furthermore, a lack of physical dynamic motions and fluid flow in cell-based surrogates may produce considerable discrepancy with in vivo responses.

### Static 3D cell culture models

Recently highlighted 3D organoid culture models have provided improved physiological cell morphology with self-organized microarchitecture [41], by which some organoid models have been utilized for demonstrating the infection of pathogenic bacteria [42] or viruses [43]. However, the nature of organoid cultures that requires heavy extracellular matrix (ECM) may hamper the effective application of NPs. For instance, organoid microenvironment can alter the result of cytotoxicity [44], where the ECM holding organoid bodies hinders the penetration of NPs into the human hepatic (HepaRG) cells because of the dense accumulation of NPs in the ECM [45] limits NP-cell interactions [46]. A recent study demonstrates that the penetration of polyethylenimine (PEI)-coated superparamagnetic NPs containing siRNA into 3D fibroblast (NIH-3T3) organoids is much slower than that of NPs into 2D cultures of the same cells, suggesting that the 3D microstructure of organoids better mimic the in vivo circumstance during the test of NP-guided siRNA gene silencing [47]. The neuroblastoma (SH-SY5Y) organoids models are also used to assess the chemotherapeutic efficacy of doxorubicin in conjugation with the NPs synthesized by the borate-containing chitosan (monomer, *N*-3-acrylamidophenylboronic acid) [48]. Interestingly, the iRGD (internalizing l-arginine, glycine and l-aspartic acid)-conjugated NPs shows significantly improved penetration and the chemotherapeutic efficiency on the multicellular organoids compared to doxorubicin alone or non-conjugated NPs alone. A prostate cancer organoid model using the prostate adenocarcinoma line (LNCaP cells) also confirms that the uptake of doxorubicin-loaded NPs induces apoptotic responses [49]. Furthermore, this model also reveals that the engineered organoids demonstrate a higher level of multidrug resistance (MDR) proteins such as multidrug resistance protein 1 (MRP1) and lung resistance-related protein (LRP), which are generally overexpressed in tumor cells due to their hypoxic condition, low nutrients supply, or low pH. In this study, 3D organoid models show significantly (*p* < 0.05) increased level of MDR compared to that obtained in the 2D cultures, suggesting that the tumor organoid models may be useful to better predict the therapeutic responses of NP-conjugated drugs in the presence of MDR effects. However, 3D organoid models still need further optimizations such as the size and density of cell aggregates and the treatment period of NPs as well as drug-conjugated NPs [50].

**Fig. 2:**
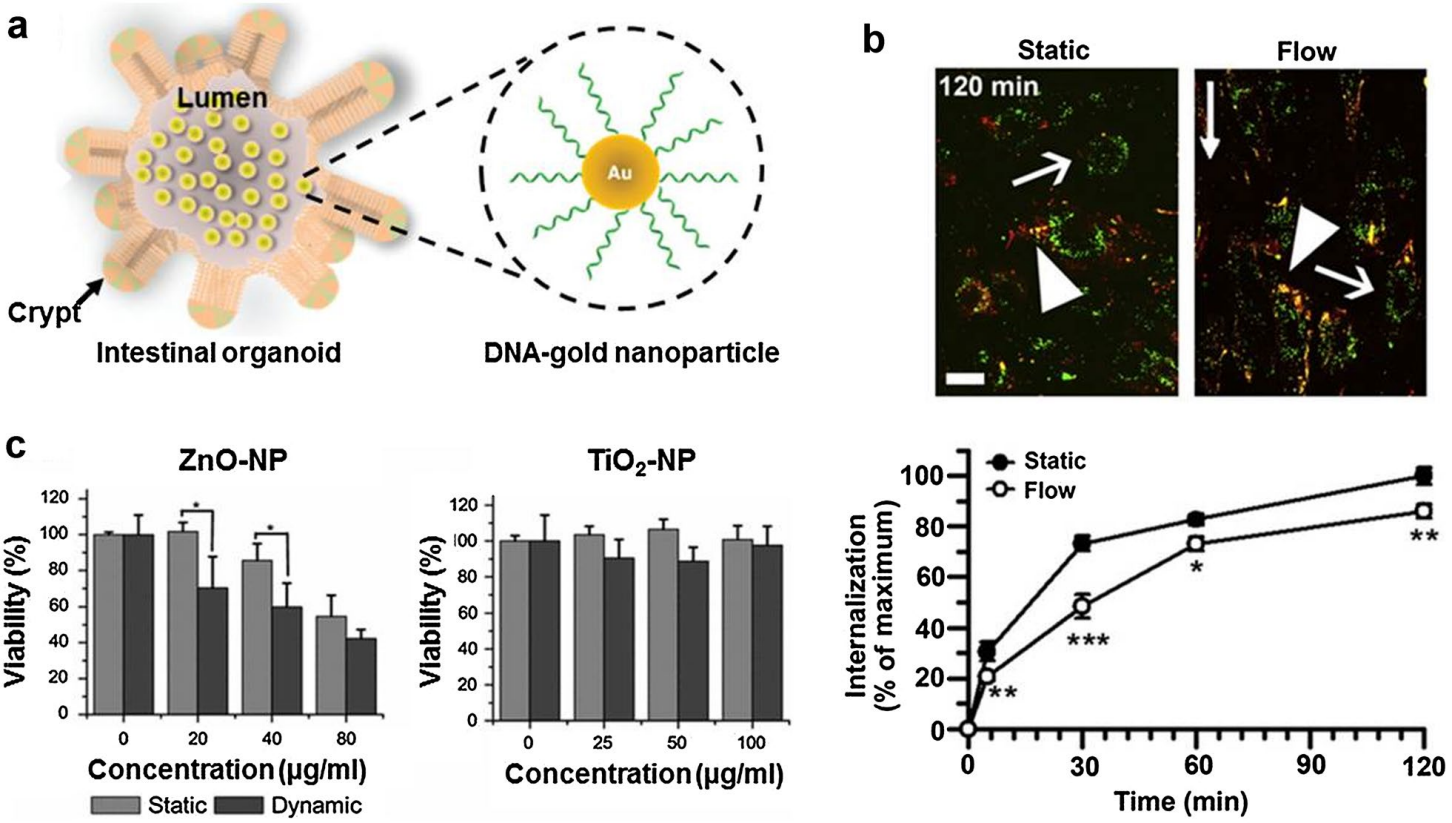
Reactivity of NPs under physiological fluidic conditions. **a** A “Trojan horse” delivery of Au-NPs conjugated with poly-thymine encapsulated inside the mouse intestinal stem cell-derived organoids. Reproduced with permission [51]. Copyright 2015, Royal Society of Chemistry. **b** Effect of shear stress on the uptake of anti-ICAM-1/NPs by endothelial cells experienced with static (i.e. no flow) or fluidic condition (shear stress, 4 dyne/cm^2^) at 37 °C for 2 h with TNF-α-containing culture medium. *Arrows* indicate the internalized FITC-labeled anti-ICAM-1/NPs (*green*) in the HUVEC. *Arrowheads* show the non-internalized anti-ICAM-1/NPs (*red*). A *graph* shows the quantification of internalized anti-ICAM-1/NPs under static versus fluidic conditions. *Scale bar* 10 µm. *p < 0.05, **p < 0.01, ***p < 0.001. Reproduced with permission [63]. Copyright 2011, Elsevier. **c** Toxicity test of ZnO and TiO_2_ NPs against lung cancer A549 cells in static and fluidic conditions (10 dyne/cm^2^). *p < 0.05. Reproduced with permission [19]. Copyright 2015, Springer

The NPs conjugated with poly-thymine DNA are also exploited for the targeted delivery using the intestinal organoids as an intervention against inflammatory bowel disease (IBD) [51] (Fig. 2a). The key idea in this study is to utilize the mouse primary intestinal stem cell (ISC)-derived intestinal organoids containing the DNA-conjugated gold NPs as a delivery vehicle (named “Trojan horse system”) to inject back into the host body, by which the negatively charged DNA-Au NPs intervene the positively charged proteins such as transferrin prevalently found in the inflamed intestine in IBD patients [52]. Using this model, improved distribution, local drug concentration, and therapeutic efficacy are expected; however possible attack of immune system when using the donor cells implanted to the recipients should be further considered.

### Microfluidic cell culture models

Microfluidics technology has provided a controllable microenvironment in micro-scale channels, in which human cells, predominantly epithelium or endothelium, can robustly and reproducibly grow under trickling slow flow of culture medium [53]. In this simple laminar flow regime, cells growing in the microchannel can communicate with each other via diffusion [54]. Microfluidic approach is valuable to assess the permeability of NPs into the neovascularized region in the tumor, by which the fluidic nature in the device mimics the in vivo vasculature with fluid shear stress [55]. Endothelial permeability of NPs has been used to validate the effective delivery of NPs to the tumor tissue. Since the vascular walls nearby the cancer lesion have nano-sized gaps, where tumor endothelial cells have ruffled margins and fragile cytoplasmic projections creating openings or small intercellular gaps in the vessel wall [56], an endothelial monolayer in this region is more permeable than that in healthy sites. Inspired by this observation, a microfluidic system lined by endothelium has been utilized to test the transport of NPs that more closely mimic the fluidic nature [57]. To evaluate the vascular permeability of NPs, human umbilical vein endothelial cells (HUVEC) have been widely used to test various NPs such as mesoporous silica NPs, gold NPs, quantum dots, and liposomes [58–62]. For the effective delivery of NPs to the endothelial cells, intercellular adhesion molecule 1 (ICAM-1) can be targeted [59], where the delivery of NPs onto the endothelial cell surface can be different in static versus microfluidic conditions (Fig. 2b) [63]. The NPs conjugated with anti-ICAM-1 antibody are less internalized into the endothelium under flow (Fig. 2b, “Flow”) compared to the cells cultured in the same condition but under static condition (Fig. 2b, “Static”), suggesting that the fluid flow-mediated shear stress may regulate the endothelial endocytosis of the delivery of NPs, partially because the reorganization of actin stress fibers under fluidic conditions may interfere the uptake of anti-ICAM-1-NPs.

For the toxicological test of NPs, the shear stress during physiological flow is also an important factor in terms of toxicity evaluation towards human cells. A microfluidic system employing an endothelial layer mimics the blood vessel [58], where introduction of gold NPs into the microfluidic channel lined by an endothelial monolayer of HUVEC shows significantly reduced cytotoxicity (*p* < 0.05) of HUVEC cells under the physiological flow at 5 µL/min (corresponding shear stress, 0.1 dyne/cm^2^) for 48 h compared to the toxicity level measured in a static 24-multiwell plate. Another study demonstrates how the defined physiological flow (shear stress, 10 dyne/cm^2^) emulating the microenvironment of lung alveolar vasculature influences the cytotoxicity of the different type of NPs on the human lung alveolar epithelial cells (A549 line) and mouse embryonic fibroblast (NIH-3T3 line) (Fig. 2c) [19]. Briefly, when the lung epithelial A549 cells are exposed to the zinc oxide (ZnO) NPs in either static or fluidic condition, the cellular toxicity in response to the fluidic versus static conditions is distinct with a statistical significance (*p* < 0.05) (Fig. 2c, “ZnO-NP”). However, cells exposed to titanium dioxide (TiO_2_) NPs do not significantly respond to TiO_2_ NPs nor to the hydrostatic conditions regardless of the concentration of NPs (Fig. 2c, “TiO_2_-NP”), suggesting that the reactivity of NPs in response to the applied shear stress can be different from the material of NPs.

In addition, photosensitizer (IR-780)-encapsulated NPs are investigated for the efficient delivery and therapeutic efficacy of NP to human lung cancer cells under microfluidic condition [64]. Human lung alveolar epithelial A549 line and normal lung fibroblast cell line (MRC-5) cultured in each PDMS/glass hybrid channel are used to quantitate the efficiency of cellular internalization of NPs at different sizes and surface properties. After the IR-780-NPs are introduced to both cancer and non-cancerous cells, energy from light-emitting diode (LED) is irradiated. The cellular uptake of NPs conjugated with IR-780 is significantly higher in the A549 cancer cells than in the MRC-5 normal lung cells, suggesting that the synthesized NPs can be used as a potential delivery system for hydrophobic photosensitizers to target the lung carcinoma.

As discussed, application of NPs in the microfluidic system is a promising way to validate the functionality of NPs in tunable and scalable controls. The conventional microfluidic approaches are robust and straightforward, and can easily be utilized to build a single microchannel with various geometric patterns [53]. However, these models mostly rely on controlling the microfluidic regimes without other microenvironmental factors such as mechanical deformations or 3D transmural tissue–tissue interface [65, 66]. Next section, we discuss how these limitations in conventional microfluidic models are improved in human organs-on-chips models to assess the functionality of NPs.

### Microengineered human organs-on-chips models

The human organs-on-chips are recently emerged microphysiological systems that are leveraging microfluidic technology. Organs-on-chips models primarily focus on the reconstitution of 3D microstructure and tissue–tissue interface of a human organ, by which they recapitulate organ-level functions related with homeostasis or pathophysiological responses. Application of organs-on-chips models have dramatically increased in drug screening and delivery [67]. In particular, new microphysiological models of human organs have emulated physiological functions of the breathing motions in the lung [65], host-microbe ecosystem and peristalsis-like mechanics in the gut [66, 68], blood cleansing functions in the spleen [69, 70], reabsorption and transport in the kidney [71, 72], microvascular networks and blood perfusion [73], or blood–brain-barrier in the brain [74, 75] that can be used to potentially validate the efficacy, targeted delivery, PK/PD profiles, functionality, and toxicity of NPs [65, 67]. While the potential of converging the NPs with organs-on-chips microsystems is enormous, applications of NPs in the existing organs-on-chips are currently nascent.

**Fig. 3:**
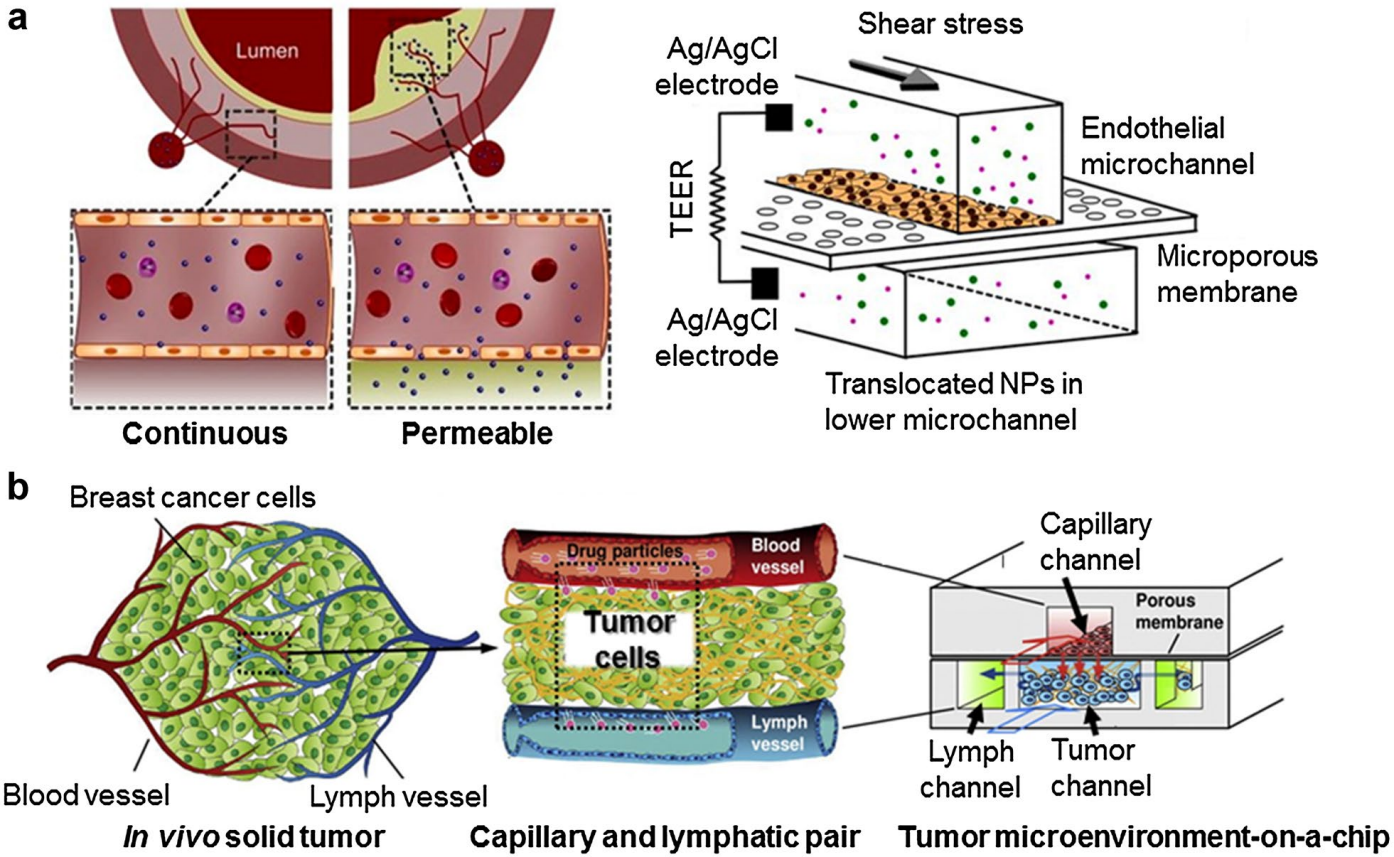
Human organs-on-chips for assessing the transport efficiency of NPs. **a** A schematic of the normal capillaries surrounding the vessel wall and the atherosclerosis microenvironment with surrounding permeable vasculature (*left*). A schematic of a microfluidic device (*right*) in which the NPs are introduced into the upper microchannel, and the translocation of NPs is measured in the lower microchannel. Reproduced with permission [76]. Copyright 2014, National Academy of Sciences. USA. **b** Schematics of the in vivo solid tumor (*left*), tumor microenvironment composed of capillary and lymphatic microvessels (*middle*), and the tumor microenvironment-on-chip (*right*). NPs are introduced into the capillary channel and they transport to the lymphatic vessels through the tumor channel, mimicking the permeable microvessels in atherosclerosis. Reproduced with permission [77]. Copyright 2014, Elsevier

Most abundantly investigated topic is the transport assessment of NPs in a 3D microfluidic organs-on-chips system. For example, a double-layered microfluidic device lined by an endothelial monolayer mimics a microvascular layer that can be utilized to study the translocation of gold NPs conjugated with poly(lactic-co-glycolic acid) (PLGA) or lipid-PLGA NPs in the atherosclerosis model with controllable permeability (Fig. 3a) [76]. The translocation of NPs across the endothelium (HUVEC) is shown to be dependent on the microvascular permeability. When the endothelial cells grown on the nanoporous membrane are challenged to the proinflammatory cytokine involved in the pathogenesis of atherosclerosis, microvascular permeability increases, which results in the increased translocation of the lipid-polymer NPs through the endothelial layer. This approach improves the physiological relevance by recapitulating the tissue–tissue interface of a local vascular microenvironment under in vivo relevant shear flow, which makes a considerable difference compared to the conventional microfluidic approach.

NPs also have been applied in the microfluidic 3D tumor model (“Tumor microenvironment-on-chip”) reconstituting the breast cancer microenvironment surrounded by the lymphatic and capillary microvessels (Fig. 3b) [77]. This model has four segregated microchannels separated by the nanoporous membrane, where each channel mimics capillary, lymph, and tumor compartments. In the upper microchannel, capillary human microvascular endothelial cells (HMVEC) are cultured on a porous membrane. In the bottom layer, there are three microchannels, in which the center channel simulates the solid tumor by employing the breast cancer cell line (MCF-7) in collagen matrix. Beside the center channel, there are two side channels mimicking the lymphatic vessels. This study reveals that the transport of NPs is dependent on the diameter of NPs (best in 100 nm), cut-off size (best in 400 nm), ECM concentrations (best in 6 mg/mL), and interstitial fluid pressure (best in 20 mmHg).

Finally, human lung-on-a-chip microfluidic device recapitulating the breathing motion is used to assess the transcytosis of NPs and their interaction with the human lung cells [65]. This microphysiological system has two juxtaposed parallel microchannels separated by a porous membrane coated with ECM (collagen or fibronectin) and lined by the human alveolar epithelial cells and capillary endothelial cells on each side. Besides the cell microchannels, there are two hollow vacuum chambers connected to the computer-controlled pneumatic system to exert cyclic rhythmical deformations. This repeated stretching emulates the breathing motions in the alveolar-capillary tissue interface, by which the transport of silica NPs (12 nm in diameter) or carboxylated Cd/Se quantum dots (16 nm in diameter) from the alveolar side (i.e. upper microchannel) to the capillary side (i.e. lower microchannel) is significantly enhanced compared to the static control. Transcytosed NPs considerably increase the proinflammatory responses of epithelial tissue and the intracellular level of reactive oxygen species (ROS) on the alveolar epithelium. A notable discovery here is that the absence of mechanical physical motions in the culture system (e.g. static Transwell culture) does not induce any detectable levels of cellular responses such as inflammatory responses or ROS generation in response to the treatment of NPs, suggesting that the assessment of the safety and efficacy of NPs and their conjugates should be potentially performed under physiological organ-specific dynamics.

## Future perspectives

**Fig. 4:**
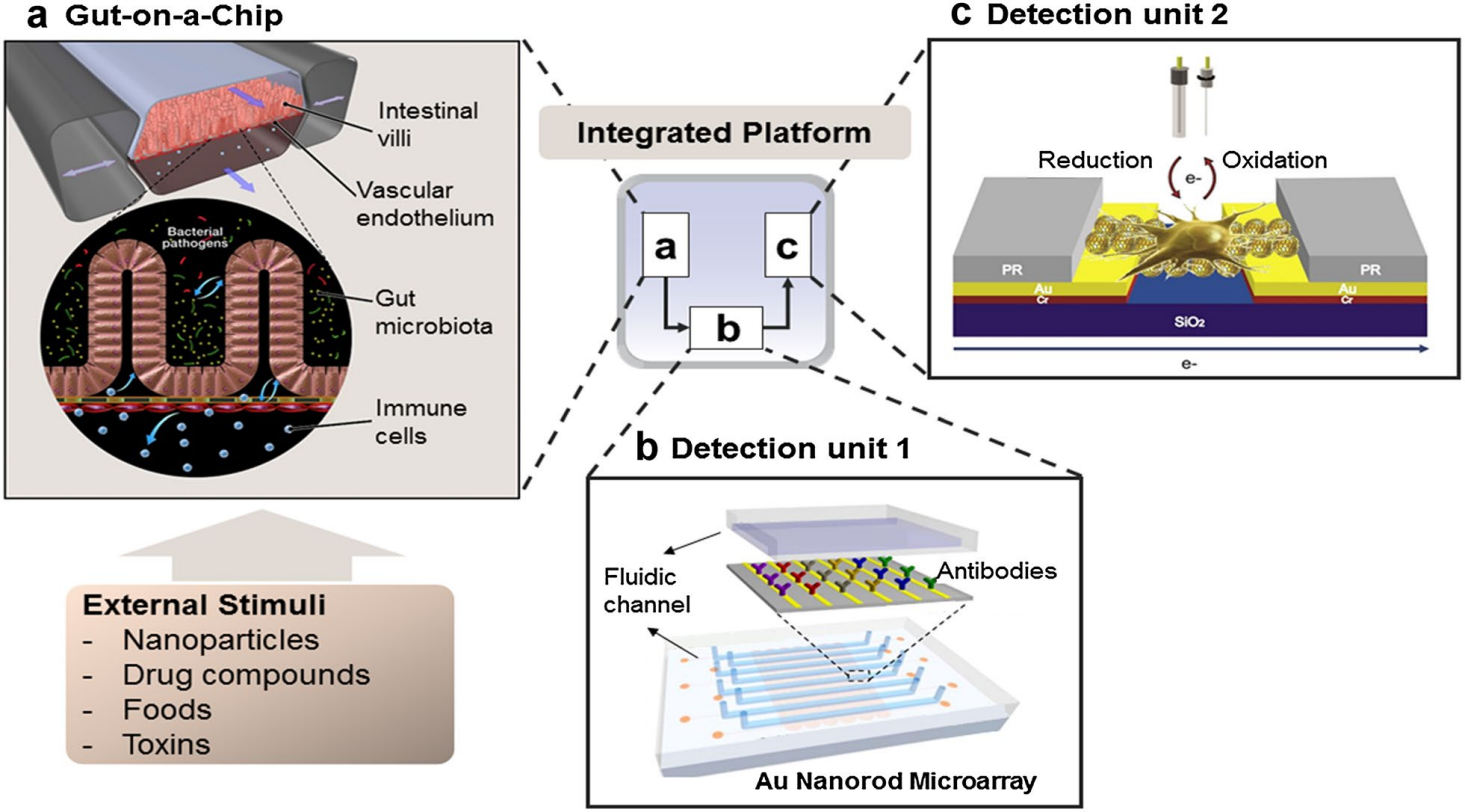
A human organ-on-a-chip microphysiological system integrated with sequential NP-guided detection systems. **a** A cross-sectional view of a human gut-on-a-chip where complex interactions of gut microbiome, epithelium, and immune components occur. Reproduced with permission [21]. Copyright 2016, Elsevier. **b** “Detection unit 1” for the on-line monitoring of cellular and microbial secretomes in the gold nanorod-deposited microfluidic channel. Reproduced with permission [86]. Copyright 2015, American Chemical Society. **c** “Detection unit 2” sequentially connected to the “Detection unit 1” to validate the differentiation of neural stem cells mimicking the physiological interaction of gut-brain axis using graphene oxide-encapsulated gold NP-guided electrochemical detection. Reproduced with permission [88]. Copyright 2013, Elsevier

The convergence of nanotechnology and bioengineering is one of the promising pathways for pharmaceutical and clinical applications. The validation of in vivo functionality and safety of NPs, however, remains as a key missing piece, which restricts the applications of NPs for the smart drug delivery or the therapeutic treatment to the patients. Furthermore, a more sophisticated integration of multiple NP-based sensing modules that can detect, amplify, and process the cellular secretomes during cell–cell or cell-microbe interactions is a critical unmet need. To surmount these challenges, organs-on-chips technology can be contemplated as an integrative platform not only to investigate the functionality of NPs on the specific organ/disease model (Fig. 3) but also to analyze diverse cellular and molecular signals in situ during the biological (e.g. host-microbe crosstalks) or physical perturbations (e.g. peristalsis-like deformations in the gut) by sequentially interconnecting the sensing and screening units (Fig. 4).

For example, a human gut-on-a-chip device [66, 68, 78] employing intestinal villi colonized by gut microbiome demonstrates complex host-microbiome interactions that can modulate epithelial differentiation and immune modulation (e.g. balance of helper vs. regulatory T lymphocytes) (Fig. 4a) [78]. During the complex host-microbe interactions, gut microbiome, intestinal epithelium, and resident immune components produce various molecular secretomes such as short-chain fatty acids (SCFA) [79], cytokines and chemokines [80], and antimicrobial peptides [81] under homeostatic condition. When this microenvironment is challenged by the factors that can induce inflammatory responses such as dysbiosis [82], susceptible genetic background of the host [83], pathogenic infection [84], or ingestion of toxic compounds [85], intestinal homeostasis is immediately compromised resulting in the switch-on of inflammatory signaling cascades and subsequent secretion of proinflammatory cytokines that can exacerbate the disease symptoms. Hence understanding the fate of those intermediate compounds and their steady-state homeostatic level is of great importance to interrogate cellular and microbial signatures in situ. And obviously, this is a critical feature that cannot be easily achieved by existing animal, 2D in vitro, and 3D static organoid models.

We anticipate sequential integration of NP-guided “Detection units” that can rapidly and accurately quantitate the transient level of metabolites and secretomes released by the cells grown in an organ chip (Fig. 4b, c). The concentration of secretomes may be instantly measured by the microfluidic SPR microarray detection unit that is linked to the gut-on-a-chip (Fig. 4b) [86]. In addition, we may assess the differentiation level of neural stem cells in the brain tissue that are communicating with the gut microbial metabolites (e.g. SCFAs) to demonstrate physiological functions of the gut-brain axis [87] using the NP-based electrochemical sensing system with in situ monitoring (Fig. 4c) [88]. Similar approaches employing the sequential integration of other organs-on-chips and NP-guided detection units will provide ample opportunities in diagnostics to understand the biological processes during the physiological reactions with spatiotemporal resolution.

In this review article, we discussed the recent progresses of human organs-on-chips microphysiological system as an alternative model that can potentially replace existing animal and in vitro cell culture models. In the current stages, there are considerable shortcomings in organs-on-chips technologies that are still challenging to provide the macroscopic feature of organ physics or inter-organ interactions. Furthermore, some features may never be possible to be realized on-chip, such as the demonstration of human behavior on-chip. However, the integration of nanotechnology with the organs-on-chips technology will provide a paradigm-shift in diagnostics and therapeutics, which can in part accelerate the practical advances for biomedical, pharmaceutical, and clinical applications.
